# Expression of CDK1^Tyr15^, pCDK1^Thr161^, Cyclin B1 (Total) and pCyclin B1^Ser126^ in Vulvar Squamous Cell Carcinoma and Their Relations with Clinicopatological Features and Prognosis

**DOI:** 10.1371/journal.pone.0121398

**Published:** 2015-04-07

**Authors:** Zhihui Wang, Ana Slipicevic, Mette Førsund, Claes G. Trope, Jahn M. Nesland, Ruth Holm

**Affiliations:** 1 Department of Pathology, The Norwegian Radium Hospital, Oslo University Hospital, Oslo, Norway; 2 Department of Obstetrics and Gynecology, The Norwegian Radium Hospital, Oslo University Hospital and University of Oslo, Oslo, Norway; 3 Department of Pathology, The Norwegian Radium Hospital, Oslo University Hospital and University of Oslo, Oslo, Norway; The University of Hong Kong, CHINA

## Abstract

Cyclin B1-CDK1 complex plays an important role in the regulation of cell cycle. Activation of Cyclin B1 and CDK1 and the formation of the complex in G2/M are under multiple regulations involving many regulators such as isoforms of 14-3-3 and CDC25 and Wee1. Abnormal expression of Cyclin B1 and CDK1 has been detected in various tumors. However, to our knowledge no previous study has investigated Cyclin B1 and CDK1 in vulvar cancer. Therefore, we evaluated the statuses of CDK1^Tyr15^, pCDK1^Thr161^, Cyclin B1 (total) and pCyclin B1^Ser126^ in 297 cases of vulvar squamous cell carcinomas by immunohistochemistry. Statistical analyses were performed to explore their clinicopathological and prognostic values. In at least 25% of tumor cases high expression of CDK1^Tyr15^, pCDK1^Thr161^, Cyclin B1 (total) and pCyclin B1^Ser126^ was observed, compared to the low levels in normal vulvar squamous epithelium. Elevated levels of CDK1^Tyr15^, pCDK1^Thr161^, Cyclin B1 (total) and pCyclin B1^Ser126^ were correlated with advanced tumor behaviors and aggressive features. Although CDK1^Tyr15^, pCDK1^Thr161^, Cyclin B1 (total) and pCyclin B1^Ser126^ could not be identified as prognostic factors, combinations of (pCDK1^Thr161 C+N^ + 14-3-3σ^N^), (pCDK1^Thr161 C+N^ + 14-3-3η^C^), (pCDK1^Thr161 C+N^ + Wee1^C^) and (pCDK1^Thr161 C+N^ + 14-3-3σ^N^ + 14-3-3η^C^ + Wee1^C^) were correlated with disease-specific survival (*p* = 0.036, *p* = 0.029, *p* = 0.042 and *p* = 0.007, respectively) in univariate analysis. The independent prognostic significance of (pCDK1^Thr161 C+N^ + 14-3-3σ^N^ + 14-3-3η^C^ + Wee1^C^) was confirmed by multivariate analysis. In conclusion, CDK1^Tyr15^, pCDK1^Thr161^, Cyclin B1 (total) and pCyclin B1^Ser126^ may be involved in progression of vulvar squamous cell carcinoma. The combination of pCDK1^Thr161^, 14-3-3σ, 14-3-3η and Wee1 was a statistically independent prognostic factor.

## Introduction

Vulvar carcinoma accounts for 3–5% of all female genital cancers with 27,000 new diagnosed patients worldwide each year [1,2]. Although vulvar cancer occurs most frequently in women above age of 65 [3,4], an increasing incidence has recently been observed among younger women [5–11]. Vulvar squamous cell carcinoma (VSCC) is the most common histological subtype, accounting for more than 80% of the cases [12]. For the last two decades, radical surgery has been the standard treatment for most patients but it is associated with high treatment-related morbidity. In recent years, less invasive novel treatments have been introduced; unfortunately a significant improvement in survival has not been achieved yet [6,13]. Therefore, identification of new biomarkers and potential therapeutic targets is highly warranted.

Cyclin B1-CDK1 complex plays an important role in G2/M cell cycle. Activation of CDK1 protein kinase and formation of Cyclin B1-CDK1 complex is an obligate step for entry into mitosis and hence is under extensive regulations [14,15]. Activity of CDK1 is controlled through association with Cyclin B1, reversible phosphorylation [16] and subcellular localizations [14,17]. Throughout the early phases of the cell cycle, inhibitory phosphorylation of CDK1 on Tyr^15^ and Thr^14^ by Wee1 and MYT1 keeps it in inactive state [15]. In late G2, CDK1 is activated by CDC25C phosphatase through dephosphorylation upon both Thr^14^ and Tyr^15^ residues, as an obligate step for the G2/M transition [18–21]. To reach maximum activity of CDK1, the phosphorylation of Thr^161^ residue by CDK1 activating kinase (CAK) is a requirement [15,22]. Therefore, dephosphorylation upon Tyr^15^ of CDK1 (CDK1^Tyr15^) and phosphorylation upon Thr^161^ of CDK1 (pCDK1^Thr161^) are both activated forms. For Cyclin B1, the residues Ser^126^ and Ser^128^ on the N-terminal are among the first ones which are autophosphorylated by Cyclin B1-CDK1 complex [17], so that pCyclin B1^Ser126^ is regarded as the equivalent form of active Cyclin B1.

Abnormal expression of Cyclin B1 and/or CDK1 has been reported in several types of tumors, such as epithelial ovarian cancer [23], non-small-cell lung cancer [24,25], tongue cancer [26], breast cancer [27], gastric cancer [28] and colorectal cancer [29]. In some of these reports the altered level of Cyclin B1 and/or CDK1 expression indicated a poor outcome of patients [25,27]. To our knowledge, no previous study has investigated CDK1 and Cyclin B1 in vulvar carcinomas. Thus, we examined the expression of CDK1^Tyr15^, pCDK1^Thr161^, Cyclin B1 (total) and pCyclin B1^Ser126^ proteins in a large cohort of VSCC and explored their clinicopatological and prognostic values. Previously in the same patient population we have identified several checkpoint proteins involved in G2/M regulations, including isoforms of CDC25 [30] and 14-3-3 [31,32] and Wee1 [33]. We evaluated the relationships of these G2/M pathway regulators together and explored combinations which might help to predict the outcome of patients with VSCC.

## Methods

### Patient materials

A retrospective study including 297 patients with VSCC who had undergone surgery at The Norwegian Radium Hospital between 1977 and 2006 was performed. The median age of patients at diagnosis was 74 years (range, 35–96 years). Nine patients received pre-surgery treatment, six of which were treated with radiotherapy and the other three with radiotherapy/chemotherapy. Radical vulvectomy was performed on 192 (65%) patients, whereas 105 (35%) patients were subjected to non-radical surgery. Seventy patients received postoperative treatment including 3 patients given chemotherapy, 63 treated with irradiation, and 4 received combination of irradiation and chemotherapy. All the patients were followed until death occurred or 5 years after study inclusion. Of the 297 patients, 100 (34%) died of vulvar cancer within 5 years after inclusion.

The tumour stage examination was performed according to the 2009 International Federation of Gynaecology and the Obstetrics (FIGO) classification system [34]. The histological re-examination of all cases was performed by one of the authors (J.M.N) according to World Health Organization recommendations [35]. Two hundred and eighty (94%) tumors were keratinizing/nonkeratinizing, 13 (5%) were basaloid and 4 (1%) were veruccoid. Normal vulva samples were obtained as controls from 10 patients operated for benign gynaecological diseases.

### Ethics statement

The approval of the study was granted by The Regional Committee for Medical Research Ethics South of Norway (S-06012), The Data Inspectorate (04/01043) and The Social and Health Directorate (04/2639 and 06/1478). In this study we have used paraffin embedded tumor tissue from vulvar cancer patients diagnosed between 1977 and 2006. Many of these patients are either dead or very old. Therefore, we have not been able to obtain patient consent. Permission has been obtained from The Social and Health Directorate (04/2639) to perform this study without patient consent.

### Immunohistochemistry

Three-μm sections made from formalin-fixed paraffin embedded tissues were immunostained using the Dako EnVision + system (K8012, Dako Cooperation, CA, USA) and DAKO Autostainer. Deparaffinization, rehydration and target retrieval were performed in a Dako PT-link and EnVision Flex target retrieval solution with high pH for pCDK1^Thr161^ and low pH for pCyclin B1^Ser126^, Cyclin B1 (total) and CDK1^Tyr15^. Endogenous peroxidase was blocked using Dako blocking reagent for 5 minutes followed by incubation with primary antibodies against pCyclin B1^Ser126^ (rabbit polyclonal antibody, AP3078a, 1:400, 62.5μg Ig/ml, Nordic BioSite AS, Täby, Sweden), Cyclin B1 (total) (rabbit monoclonal antibody, Clone Y106, 1:700, 0.45μg IgG/ml, Epitomics Inc., CA, USA), pCDK1^Thr161^ (rabbit polyclonal antibody, sc-101654, 1:100, 1μg IgG/ml, Santa Cruz Biotechnology Inc., Santa Cruz, CA, USA) and CDK1^Tyr15^ (rabbit monoclonal antibody, Clone: 6k113, 1:1000, 0.1μg IgG/ml, United States Biological Inc., MA, USA). Thereafter, the sections were incubated with Dako EnVision FLEX+ rabbit linker for 15 minutes followed by incubation with Dako EnVision FLEX/HRP for an additional 30 minutes. For visualization of staining, the sections were treated with 3'3-diaminobenzidine tetra-hydrochloride (DAB), counterstained with haematoxylin and mounted in Richard-Allan Scientific Cytoseal XYL (Thermo Scientific, Waltham, MA, USA).

All of the sample series had appropriate positive controls including tonsil [pCyclin B1^Ser126^ and Cyclin B1 (total)] and placenta (pCDK1^Thr161^ and CDK1^Tyr15^). Negative controls included substitutions of (i) polyclonal anti-pCyclin B1^Ser126^ (ii) polyclonal anti-pCDK1^Thr161^ and (iii) monoclonal anti-Cyclin B1 (total) and CDK1^Tyr15^ with (i) normal rabbit Ig (ii) normal rabbit IgG and (iii) normal rabbit IgG monoclonal at the same concentration as the primary antibodies.

The immunohistochemical staining was evaluated without knowledge of the patient outcome. Semi-quantitative classes were used to describe the immunostaining. The extent of staining was scored into 4 levels regarding percent of positive tumor cells (absent, 0; < 10%, 1; 10–50%, 2; > 50%, 3), while staining intensity was scored into 4 levels as well (absent, 0; weak, 1; moderate, 2; strong, 3). Immunoreactivity in cytoplasm and nucleus was calculated separately by multiplying the scores of the staining extent and intensity of each slide, and composite scores were ranged from 0 to 9. High protein level was defined when composite score > 3 and low when composite score ≤ 3. The cutoff values for the immunostaining were based on staining pattern observed in normal vulvar epithelium.

### Statistical analyses

The Pearson’s chi-square (χ^2^) test was performed to evaluate the relationship between the expression of CDK1 (CDK1^Tyr15^ and pCDK1^Thr161^) and Cyclin B1 (total and pCyclin B1^Ser126^) and clinicopathological parameters. Survival analyses were evaluated on the whole group of 297 VSCC and on the group of 288 VSCC which excluded nine patients who have received pre-surgery treatment. Kaplan and Meier method was used to calculate the disease-specific survival from the date of diagnosis to vulvar cancer related death. Survival rate comparison was performed by the log-rank test. Univariate and multivariate evaluation of survival were calculated by using Cox proportional hazards regression. Patients were censored after 5 years. In the multivariate analysis, a backward stepwise regression with a *p* value of 0.05 as the inclusion criterion was used. All analyses were executed by using SPSS 18.0 statistical software package (SPSS, Chicago, IL, USA). Statistical significance was considered as *p* ≤ 0.05.

## Results

### CDK1^Tyr15^, pCDK1^Thr161^, Cyclin B1 (total) and pCyclin B1^Ser126^ proteins expression

**Fig 1:**
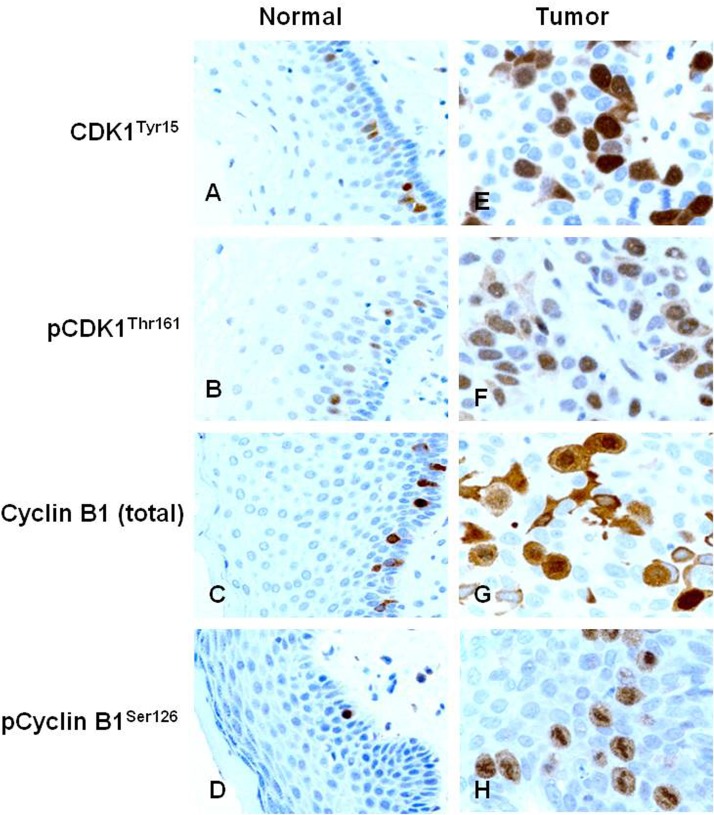
Expression of CDK1^Tyr15^, pCDK1^Thr161^, Cyclin B1 (total) and pCyclin B1^Ser126^ in vulvar squamous epithelium. Immunostaining of (A) CDK1^Tyr15^, (B) pCDK1^Thr161^, (C) Cyclin B1 (total) and (D) pCyclin B1^Ser126^ in normal vulvar epithelium (х300) and (E) CDK1^Tyr15^, (F) pCDK1^Thr161^, (G) Cyclin B1 (total) and (H) pCyclin B1^Ser126^ in VSCC (х600).

In 10 cases of normal vulvar squamous epithelium, immunoreactivity of CDK1^Tyr15^, pCDK1^Thr161^, Cyclin B1 (total) and pCyclin B1^Ser126^ were detected in basal and parabasal layers (score = 3 in both cytoplasm and nucleus, Fig 1A–1D).

The immunostaining results in vulvar carcinomas are summarized in S1 Table. High cytoplasmic staining (score > 3) of CDK1^Tyr15^, pCDK1^Thr161^, Cyclin B1 (total) and pCyclin B1^Ser126^ were observed in 195 (66%), 97 (33%), 208 (70%) and 73 (25%) of the cases, respectively. In the nucleus, high expression (score > 3) of CDK1^Try15^, pCDK1^Thr161^, Cyclin B1 (total) and pCyclin B1^Ser126^ were detected in 78 (26%), in 231 (78%), 82 (28%) and 75 (25%) of the cases, respectively (Fig 1E–1H). No difference of the immunostaining was observed on the front of invasion and the center of the tumor.

### Association of CDK1^Tyr15^, pCDK1^Thr161^, Cyclin B1 (total) and pCyclin B1^Ser126^ proteins with clinicopathological variables

High cytoplasmic level of CDK1^Tyr15^ and pCDK1^Thr161^ and high expression of Cyclin B1 (total) in either cytoplasm or nucleus were correlated with large tumor diameter, poor histological differentiation and deep invasion (S2 and S3 Tables). High nuclear level of pCDK1^Thr161^ and high expression of pCyclin B1^Ser126^ in either cytoplasm or nucleus were associated to younger age, high FIGO substage and poor histological differentiation. High protein level of CDK1^Tyr15^ in cytoplasm and that of pCDK1^Thr161^ in nucleus also had correlation with presence of lymph node metastasis.

### Correlations between CDK1^Tyr15^, pCDK1^Thr161^, Cyclin B1 (total) and pCyclin B1^Ser126^ and other G2/M cell cycle factors

Since our cohort of VSCC has previously been tested for isoforms of CDC25 [30] and 14-3-3 [31,32], Wee1 [33] and HPV [36], we have examined the relationship between CDK1^Tyr15^, pCDK1^Thr161^, Cyclin B1 (total) and pCyclin B1^Ser126^ and these factors (S4 and S5 Tables).

Protein levels of pCDK1^Tyr15^, pCDK1^Thr161^, Cyclin B1 (total) and pCyclin B1^Ser126^ in either cytoplasm or nucleus were positive correlated to each other. The main findings when comparing these proteins with isoforms of 14-3-3 and CDC25 and Wee1 were that i) cytoplasmic expression of CDK1^Tyr15^ and pCyclin B1^Ser126^ were positive correlated to 14-3-3η, while in nucleus high expression of CDK1^Tyr15^, pCDK1^Thr161^ and pCyclin B1^Ser126^ were correlated to high level of 14-3-3ε, ii) high protein levels of cytoplasmic CDK1^Tyr15^, pCDK1^Thr161^ and pCyclin B1^Ser126^ all related to high level of cytoplasmic pCDC25C^Ser216^ and iii) high levels of CDK1^Tyr15^, pCDK1^Thr161^ and pCyclin B1^Ser126^ in cytoplasm and nucleus were all associated with high expression of Wee1 in cytoplasm and nucleus, respectively.

### Survival

**Fig 2:**
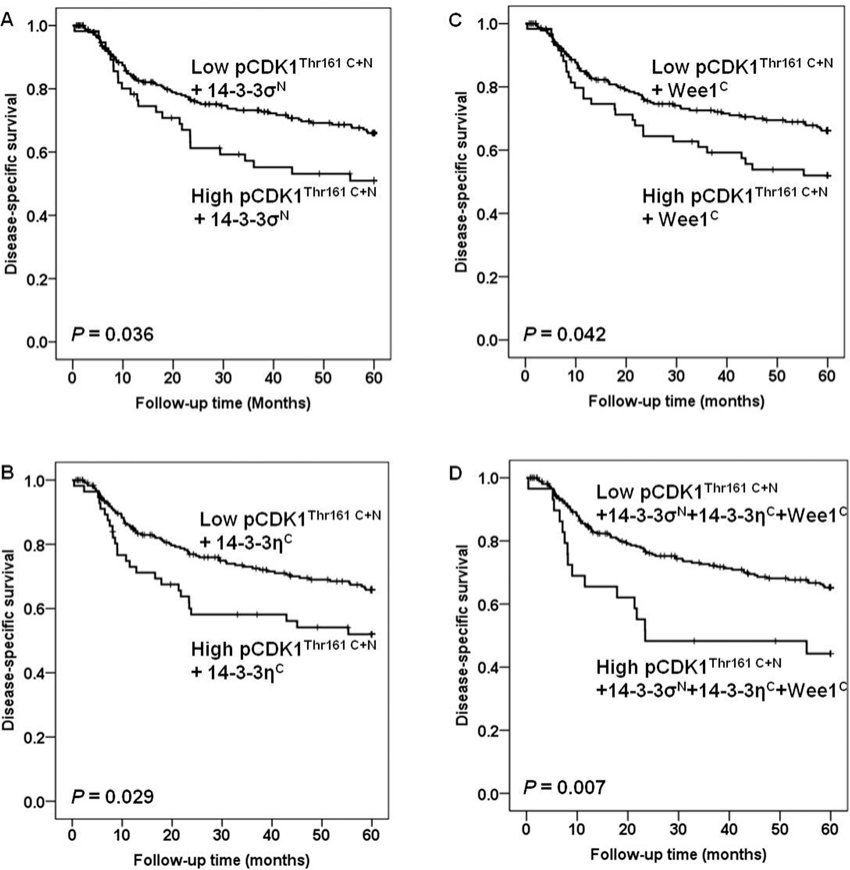
Survival curves using the Kaplan-Meier method (all 297 VSCC included). The Kaplan-Meier curves of disease-specific survival in relation to combinations of (A) (pCDK1^Thr161 C+N^ + 14-3-3σ^N^), (B) (pCDK1^Thr161 C+N^ + 14-3-3η^C^), (C) (pCDK1^Thr161 C+N^ + Wee1^C^) and (D) (pCDK1^Thr161 C+N^ + 14-3-3σ^N^ + 14-3-3η^C^ + Wee1^C^). The *p*-values differ slightly from those in Table 1 due to the use of the log-rank test as opposed to the Cox-regression analysis.

**Fig 3:**
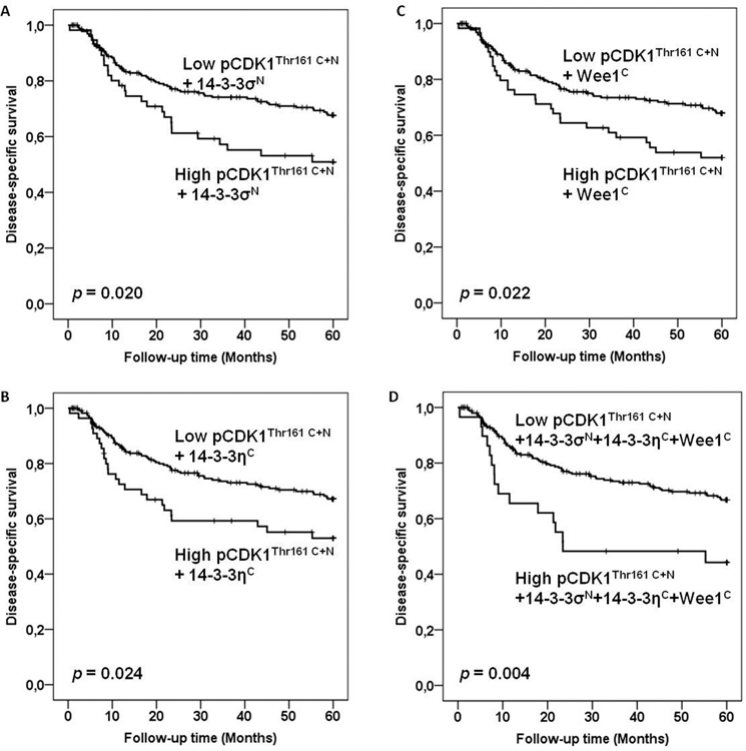
Survival curves using the Kaplan-Meier method (288 VSCC without pre-surgery treatment cases). The Kaplan-Meier curves of disease-specific survival in relation to combinations of (A) (pCDK1^Thr161 C+N^ + 14-3-3σ^N^), (B) (pCDK1^Thr161 C+N^ + 14-3-3η^C^), (C) (pCDK1^Thr161 C+N^ + Wee1^C^) and (D) (pCDK1^Thr161 C+N^ + 14-3-3σ^N^ + 14-3-3η^C^ + Wee1^C^). The *p*-values differ slightly from those in Table 2 due to the use of the log-rank test as opposed to the Cox-regression analysis.

With all 297 VSCC included, univariate analysis revealed no association between protein levels of CDK1^Tyr15^, pCDK1^Thr161^, Cyclin B1 (total) and pCyclin B1^Ser126^ in either cytoplasm or nucleus and disease-specific survival. Elevated pCDK1^Thr161^ in both cytoplasm and nucleus (pCDK1^Thr161 C+N^) showed a trend to poor disease-specific survival (*p* = 0.078). Combinations of high expression of pCDK1^Thr161 C+N^ with high levels of nuclear 14-3-3σ (14-3-3σ^N^), cytoplasmic 14-3-3η (14-3-3η^C^) or cytoplasmic Wee1 (Wee1^C^), were correlated with poor disease-specific survival (*p* = 0.036, *p* = 0.029 and *p* = 0.042, respectively) (Fig 2A–2C). Such correlation also existed between the combination of (pCDK1^Thr161 C+N^ + 14-3-3σ^N^ + 14-3-3η^C^ + Wee1^C^) and survival (*p* = 0.007) (Fig 2D). In multivariate analysis, lymph node metastases, tumor diameter, infiltration of vessel, age and combinations of (pCDK1^Thr161 C+N^ + 14-3-3σ^N^), (pCDK1^Thr161 C+N^ + 14-3-3η^C^), (pCDK1^Thr161 C+N^ + Wee1^C^) and (pCDK1^Thr161 C+N^ + 14-3-3σ^N^ + 14-3-3η^C^ + Wee1^C^) were included as important parameters. Lymph node metastases, age, tumor diameter and the combination of (pCDK1^Thr161 C+N^ + 14-3-3σ^N^ + 14-3-3η^C^ + Wee1^C^) retained independent prognostic significance for patients with VSCC (Table 1). Survival analyses among the group of 288 VSCC which excluded the nine pre-surgery cases revealed similar results (Table 2 and Fig 3A–3D).

**Table 1:** Relative risk (RR) of dying from vulvar cancer (all 297 VSCC included).

Variables	Univariate analysis			Multivariate analysis		
	RR	95% CI^a^	*p*	RR	95% CI^a^	*p*
Lymph node metastases	2.55	1.98–3.28	<0.001	2.26	1.70–3.01	<0.001
Age	1.54	1.16–2.06	0.003	1.54	1.11–2.15	0.011
Tumor diameter	1.92	1.47–2.51	<0.001	1.35	1.00–1.82	0.050
Infiltration of vessel	2.29	1.51–3.47	<0.001	-	-	-
pCDK1^Thr161^ ^C^^+^^N^^,^ ^b^ + 14-3-3σ^N^	1.61	1.03–2.52	0.037	-	-	-
pCDK1^Thr161^ ^C^^+^^N^^,^ ^b^ + 14-3-3η^C^	1.64	1.05–2.56	0.030	-	-	-
pCDK1^Thr161^ ^C^^+^^N^^,^ ^b^ + Wee1^C^	1.57	1.01–2.43	0.044	-	-	-
pCDK1^Thr161^ ^C^^+^^N^^,^ ^b^ + 14-3-3σ^N^ +14-3-3η^C^ + Wee1^C^	2.05	1.20–3.50	0.009	1.93	1.09–3.43	0.024

^a^ 95% confidence interval

^b^ High vs low expression

C = Cytoplasm

N = Nucleus

**Table 2:** Relative risk (RR) of dying from vulvar cancer (288 VSCC without neoadjuvant treatment cases).

Variables	Univariate analysis			Multivariate analysis		
	RR	95% CI^a^	*p*	RR	95% CI^a^	*p*
Lymph node metastases	2.58	1.99–3.54	<0.001	2.40	1.80–3.19	<0.001
Age	1.57	1.17–2.11	0.003	1.54	1.11–2.15	0.011
Tumor diameter	1.95	1.48–2.56	<0.001	1.47	1.09–1.98	0.011
Infiltration of vessel	2.40	1.56–3.67	<0.001	-	-	-
pCDK1^Thr161^ ^C^^+^^N^^,^ ^b^ + 14-3-3σ^N^	1.70	1.08–2.67	0.021	-	-	-
pCDK1^Thr161^ ^C^^+^^N^^,^ ^b^ + 14-3-3η^C^	1.68	1.06–2.66	0.026	-	-	-
pCDK1^Thr161^ ^C^^+^^N^^,^ ^b^ + Wee1^C^	1.67	1.07–2.59	0.024	-	-	-
pCDK1^Thr161^ ^C^^+^^N^^,^ ^b^ + 14-3-3σ^N^ +14-3-3η^C^ + Wee1^C^	2.15	1.26–3.68	0.005	1.91	1.08–3.39	0.026

^a^ 95% confidence interval

^b^ High vs low expression

C = Cytoplasm

N = Nucleus

## Discussion

In the current study, at least 25% of VSCC showed high expression of Cyclin B1^Ser126^, CDK1^Tyr15^ and pCDK1^Thr161^ in nucleus, compared to the low levels of these proteins in normal vulvar squamous epithelium. Interestingly, the high levels of these activated types of Cyclin B1 and CDK1 did not exclusively exist in nucleus; in fact there were at least 25% of VSCC with high expression of pCyclin B1^Ser126^, CDK1^Tyr15^ and pCDK1^Thr161^ in cytoplasm as well. In addition, high protein levels of pCyclin B1^Ser126^, CDK1^Tyr15^ and pCDK1^Thr161^ in either cytoplasm or nucleus were positive correlated to each other. These data suggests that in VSCC activated complex of pCyclin B1^Ser126^-CDK1^Tyr15^/pCDK1^Thr161^ can be formed in both cytoplasm and nucleus. This observation is supported by a recent study of Gavet et al. showing that as soon as Cyclin B1-CDK1 is activated in Hela cells, the complex rapidly accumulates in the nucleus, while a substantial amount of Cyclin B1-CDK1 still remains in the cytoplasm, thus mitotic events could synchronize from both cytoplasm and nucleus [17]. Taken together, our findings that high levels of pCyclin B1^Ser126^, CDK1^Tyr15^ and pCDK1^Thr161^ are observed together in at least 25% of our cases suggest that these proteins may contribute to tumorigenesis of a subset of VSCC.

Previously, conflict findings regarding connection between Cyclin B1 and malignant features have been reported. In some tumors like gastric cancer [28] and colorectal cancer [29], overexpression of Cyclin B1 was associated with less aggressive tumour behaviour. In contrast, in other tumors including oesophageal [37], gastric [38], tongue [26], breast [27] and non-small cell lung cancer [24,25], high level of Cyclin B1 was associated with aggressive tumor behavior. We found that in VSCC high expression of Cyclin B1 (total) was significantly associated with malignant features, including large tumor diameter, poor histological differentiation and deep invasion. These results indicate that the role of Cyclin B1 is cancer specific. For the first time, we have found that pCyclin B1^Ser126^, pCDK1^Thr161^ and CDK1^Tyr15^ all significantly correlated to tumor malignancy and aggressiveness of VSCC. Cytoplasmic overexpression of CDK1^Tyr15^and pCDK1^Thr161^ shared the connections to large tumor diameter, poor histological differentiation and deep invasion, while nuclear overexpression of both pCDK1^Thr161^ and pCyclin B1^Ser126^ were associated with younger age, high FIGO substage and poor histological differentiation. In summary, our findings indicate that CDK1^Tyr15^, pCDK1^Thr161^, Cyclin B1 (total) and pCyclin B1^Ser126^ may play a role in the progression of VSCC.

Comparing the activated types of CDK1 and Cyclin B1 with other G2/M cell cycle regulators we found that cytoplasmic expression of CDK1^Tyr15^ and pCyclin B1^Ser126^ were positive correlated to cytoplasmic 14-3-3η. This is in line with previous findings where 14-3-3 has been reported to sequester Cyclin B1-CDK1 complex in cytoplasm and prevent its entry into nucleus, resulting in G2/M cell cycle arrest [39,40]. Interestingly, high expression of CDK1^Tyr15^, pCDK1^Thr161^ and pCyclin B1^Ser126^ in the nucleus were correlated to high nuclear level of 14-3-3ε. One hypothesis is that 14-3-3ε, for unknown reason in VSCC, is unable to bind to pCyclin B1^Ser126^-CDK1^Tyr15^/pCDK1^Thr161^ complex and transport it out of the nucleus, thus the complex will then stay in the nucleus and trigger G2/M transition [14,15]. We also discovered that the high protein levels of cytoplasmic CDK1^Tyr15^, pCDK1^Thr161^ and pCyclin B1^Ser126^ all correlated with high level of cytoplasmic pCDC25C^Ser216^. This is in agreement with the theory that the main function of CDC25C is to activate CDK1 by removing the inhibitory phosphate groups from residues Thr^14^ and Tyr^15^, thus the active complex of pCyclin B1^Ser126^-CDK1^Tyr15^/pCDK1^Thr161^ could be formed [18–21]. Furthermore, our results showed surprisingly positive relations between Wee1 and the activated CDK1 and Cyclin B1. We found that high levels of CDK1^Tyr15^, pCDK1^Thr161^ and pCyclin B1^Ser126^ in cytoplasm and nucleus were all associated with high expression of Wee1 in cytoplasm and nucleus, respectively. Although this observation is not in agreement with previous findings in other tumor types, in those Wee1 acts as a negative regulator of CDK1 in G2/M cell cycle [25,41,42], it is consistent with our previous study [33], where the high protein level of Wee1 is correlated to tumor malignancy and aggressive phenotype in VSCC. Those data suggests that in VSCC the activation of CDK1 and Cyclin B1 is accompanied by high Wee1 expression. In our previous analysis of the same cohort of VSCC, 70% of the cases had high expression of pCDC25C^Ser216^ in nucleus. Therefore it is possible that this high activation of pCDC25C^Ser216^ is opposing inhibitory Wee1 effect, resulting in activation of CDK1 and Cyclin B1. However, reasons why Wee1 loses its inhibitory function and even rather positively relates to CDK1 in VSCC deserve further investigation.

Considering bias might be raised when survival rates were evaluated in patients with heterogeneous treatment, we performed survival analyses on the group which excluded the nine patients who have received neoadjuvant treatment, in addition to the survival analyses on the whole group of 297 VSCC. The similarity of the data from the two groups suggests that the numbers of the excluded cases are low and therefore will not change the overall survival. Our results showed that neither cytoplasmic nor nuclear expression of CDK1^Tyr15^ pCDK1^Thr161^, Cyclin B1 (total) and pCyclin B1^Ser126^ was associated with disease-specific survival in patients with VSCC. However, high expression of pCDK1^Thr161 C+N^ showed a trend to poor disease-specific survival. In esophageal cancer [43], gastric cancer [38], lymph node-negative breast cancer [44] and non-small cell lung cancer [25], overexpression of Cyclin B1 have been found to be an useful prognostic parameter. In gastrointestinal stromal tumor [45,46], CDK1 is associated with a shorter period of disease-free survival. Since the outcome of patients most likely is the result of multiple regulations of cell cycle, we performed survival analysis by combining CDK1^Thr161 C+N^ and other cell cycle regulators including 14-3-3s, CDC25s and/or Wee1. Interestingly, in univariate but not in multivariate analysis, high expression of pCDK1^Thr161 C+N^ combined with high expression of 14-3-3σ^N^ or 14-3-3η^C^ or Wee1^C^ showed a significantly correlation with disease-specific survival. Furthermore, when combining high expression of pCDK1^Thr161 C+N^ + 14-3-3σ^N^ + 14-3-3η^C^ + Wee1^C^, a significant correlation to survival was found in univariate as well as in multivariate analysis. This is in line with previous studies in ovarian cancer [23], malignant peripheral nerve sheath tumors [47] and VSCC [48], where significant correlations have been observed between some combinations of cell cycle factors and survival. In summary, our results indicate that it is important to co-analysis G2/M proteins in VSCC. We have uncovered certain combinations of G2/M regulators whose status can predict outcome of VSCC.

## Conclusion

In conclusion, the abnormal expression of pCDK1^Thr161^, CDK1^Tyr15^, Cyclin B1 (total) and pCyclin B1^Ser126^ and their associations with malignancy and aggressive phenotypes suggest that they are involved in tumorigenesis and progression of VSCC. The combinations of G2/M cell cycle regulators including pCDK1^Thr161^, 14-3-3σ^N^, 14-3-3η^C^ and Wee1^C^ may predict the survival of patients with VSCC.

## Supporting Information

S1 Table Immunostaining results for CDK1^Tyr15^, pCDK1^Thr161^, Cyclin B1 (total) and pCyclin B1^Ser126^. (DOCX)

S2 Table CDK1^Tyr15^ and pCDK1^Thr161^ expression in relation to clinicopathological variables. (DOCX)

S3 Table Cyclin B1 (total) and pCyclin B1^Ser126^ expression in relation to clinicopathological variables. (DOCX)

S4 Table CDK1^Tyr15^ and pCDK1^Thr161^ expression in relation to cell cycle proteins and HPV. (DOCX)

S5 Table Cyclin B1 (total) and pCyclin B1^Ser126^ expression in relation to cell cycle proteins and HPV. (DOCX)
