# Supplementary material for: Expression of CDK1Tyr15, pCDK1Thr161, Cyclin B1 (Total) and pCyclin B1Ser126 in Vulvar Squamous Cell Carcinoma and Their Relations with Clinicopatological Features and Prognosis
Source: PLoS One. 2015 Apr 7;10(4):e0121398. doi: 10.1371/journal.pone.0121398 (PMC4388712; doi:10.1371/journal.pone.0121398)
Supplement: S1 Table — (DOCX) [file pone.0121398.s001.docx]

**S1 Table.** Immunostaining results for CDK1^Tyr15^, pCDK1^Thr161^, Cyclin B1 (total) and pCyclin B1^Ser126^

|  |  | **CDK1^Tyr15^** | |  | **pCDK1^Thr161^** | |  | **Cyclin B1 (total)** | |  | **pCyclin B1^Ser126^** | |
| --- | --- | --- | --- | --- | --- | --- | --- | --- | --- | --- | --- | --- |
| **Score** |  | **C (%)** | **N (%)** |  | **C (%)** | **N (%)** |  | **C (%)** | **N (%)** |  | **C (%)** | **N (%)** |
| 0 |  | 6 (2.0) | 19 (6.4) |  | 58 (19.5) | 4 (1.3) |  | 0 (0) | 0 (0) |  | 16 (5.4) | 16 (5.4) |
| 1 |  | 1 (0.3) | 1 (0.3) |  | 15 (5.1) | 3 (1.0) |  | 0 (0) | 0 (0) |  | 0 (0) | 0 (0) |
| 2 |  | 8 (2.7) | 4 (1.3) |  | 119 (40.1) | 12 (4.0) |  | 0 (0) | 0 (0) |  | 0 (0) | 0 (0) |
| 3 |  | 87 (29.3) | 195 (65.7) |  | 8 (2.7) | 47 (15.8) |  | 89 (30.0) | 215 (72.4) |  | 208 (70.0) | 206 (69.4) |
| 4 |  | 20 (6.7) | 14 (4.7) |  | 81 (27.3) | 45 (15.2) |  | 3 (1.0) | 0 (0) |  | 0 (0) | 0 (0) |
| 6 |  | 143 (48.1) | 62 (20.9) |  | 13 (4.4) | 115 (38.7) |  | 157 (52.9) | 82 (27.6) |  | 72 (24.2) | 75 (25.3) |
| 9 |  | 32 (10.8) | 2 (0.7) |  | 3 (1.0) | 71 (23.9) |  | 48 (16.2) | 0 (0) |  | 1 (0.3) | 0 (0) |
| Total |  | 297 (100.0) | 297 (100.0) |  | 297 (100.0) | 297 (100.0) |  | 297 (100.0) | 297 (100.0) |  | 297 (100.0) | 297 (100.0) |

C: Cytoplasm

N: Nucleus
