# Supplementary material for: Expression of CDK1Tyr15, pCDK1Thr161, Cyclin B1 (Total) and pCyclin B1Ser126 in Vulvar Squamous Cell Carcinoma and Their Relations with Clinicopatological Features and Prognosis
Source: PLoS One. 2015 Apr 7;10(4):e0121398. doi: 10.1371/journal.pone.0121398 (PMC4388712; doi:10.1371/journal.pone.0121398)
Supplement: S2 Table — (DOCX) [file pone.0121398.s002.docx]

**S2 Table.** CDK1^Tyr15^ and pCDK1^Thr161^ expression in relation to clinicopathological variables

|  |  | **CDK1^Tyr15^** | | | | | | |  | **pCDK1^Thr161^** | | | | | | |
| --- | --- | --- | --- | --- | --- | --- | --- | --- | --- | --- | --- | --- | --- | --- | --- | --- |
| **Variables** |  | **(C)** | | |  | **(N)** | | |  | **(C)** | | |  | **(N)** | | |
|  | **No.** | **High** | **(%)** | ***p*** |  | **High** | **(%)** | ***p*** |  | **High** | **(%)** | ***p*** |  | **High** | **(%)** | ***p*** |
| Age |  |  |  | 0.517^1^ |  |  |  | 0.423^1^ |  |  |  | 0.357^1^ |  |  |  | 0.008^1^ |
| 25-69 | 117 | 77 | (66) |  |  | 35 | (30) |  |  | 42 | (36) |  |  | 100 | (85) |  |
| 70-84 | 146 | 99 | (68) |  |  | 34 | (23) |  |  | 45 | (31) |  |  | 108 | (74) |  |
| 85+ | 34 | 19 | (56) |  |  | 9 | (27) |  |  | 10 | (29) |  |  | 23 | (68) |  |
| FIGO |  |  |  | 0.058^2^ |  |  |  | 0.481^2^ |  |  |  | 0.077^2^ |  |  |  | 0.044^2^ |
| Ia | 10 | 4 | (40) |  |  | 2 | (20) |  |  | 3 | (30) |  |  | 6 | (60) |  |
| Ib | 137 | 82 | (60) |  |  | 34 | (25) |  |  | 36 | (26) |  |  | 97 | (71) |  |
| II | 13 | 9 | (69) |  |  | 3 | (23) |  |  | 5 | (39) |  |  | 11 | (85) |  |
| IIIa | 64 | 47 | (73) |  |  | 18 | (28) |  |  | 25 | (39) |  |  | 59 | (92) |  |
| IIIb | 38 | 28 | (74) |  |  | 8 | (21) |  |  | 12 | (32) |  |  | 29 | (76) |  |
| IIIc | 12 | 8 | (67) |  |  | 4 | (33) |  |  | 3 | (25) |  |  | 10 | (83) |  |
| IVa | 5 | 2 | (40) |  |  | 1 | (20) |  |  | 1 | (20) |  |  | 4 | (80) |  |
| IVb | 13 | 12 | (92) |  |  | 7 | (54) |  |  | 9 | (69) |  |  | 11 | (85) |  |
| Not available | 5 |  |  |  |  |  |  |  |  |  |  |  |  |  |  |  |
| Lymph node metastasis |  |  |  | 0.022^3^ |  |  |  | 0.717^3^ |  |  |  | 0.146^3^ |  |  |  | 0.009^3^ |
| None | 164 | 97 | (59) |  |  | 41 | (25) |  |  | 45 | (27) |  |  | 118 | (72) |  |
| Unilateral | 89 | 64 | (72) |  |  | 26 | (29) |  |  | 35 | (39) |  |  | 79 | (89) |  |
| Bilateral | 38 | 30 | (79) |  |  | 9 | (24) |  |  | 13 | (34) |  |  | 30 | (79) |  |
| Not available | 6 |  |  |  |  |  |  |  |  |  |  |  |  |  |  |  |
| Tumor diameter (cm) |  |  |  | 0.001^1^ |  |  |  | 0.993^1^ |  |  |  | 0.007^1^ |  |  |  | 0.899^1^ |
| 0.3-2.5 | 88 | 47 | (53) |  |  | 22 | (25) |  |  | 19 | (22) |  |  | 69 | (78) |  |
| 2.6-4.0 | 93 | 60 | (65) |  |  | 22 | (24) |  |  | 30 | (32) |  |  | 69 | (74) |  |
| 4.1-20.0 | 100 | 77 | (77) |  |  | 25 | (25) |  |  | 40 | (40) |  |  | 79 | (79) |  |
| Not available | 16 |  |  |  |  |  |  |  |  |  |  |  |  |  |  |  |
| Tumor differentiation |  |  |  | <0.001^3^ |  |  |  | 0.087^3^ |  |  |  | <0.001^3^ |  |  |  | 0.014^3^ |
| Well | 73 | 34 | (47) |  |  | 14 | (19) |  |  | 13 | (18) |  |  | 48 | (66) |  |
| Moderate | 153 | 101 | (66) |  |  | 39 | (26) |  |  | 45 | (29) |  |  | 123 | (80) |  |
| Poor | 71 | 60 | (84) |  |  | 25 | (35) |  |  | 39 | (55) |  |  | 60 | (85) |  |
| Depth of invasion (mm) |  |  |  | <0.001^1^ |  |  |  | 0.551^1^ |  |  |  | 0.032^1^ |  |  |  | 0.290^1^ |
| 0.0-4.0 | 76 | 39 | (51) |  |  | 22 | (29) |  |  | 18 | (24) |  |  | 57 | (75) |  |
| 4.1-8.0 | 98 | 61 | (62) |  |  | 22 | (22) |  |  | 30 | (31) |  |  | 81 | (83) |  |
| 8.1-40.0 | 112 | 86 | (77) |  |  | 27 | (24) |  |  | 43 | (38) |  |  | 83 | (74) |  |
| Not available | 11 |  |  |  |  |  |  |  |  |  |  |  |  |  |  |  |
| Infiltration of vessel |  |  |  | 0.742^3^ |  |  |  | 0.232^3^ |  |  |  | 0.816^3^ |  |  |  | 0.592^3^ |
| No | 229 | 150 | (66) |  |  | 57 | (25) |  |  | 74 | (32) |  |  | 176 | (77) |  |
| Yes | 65 | 44 | (68) |  |  | 21 | (32) |  |  | 22 | (34) |  |  | 52 | (80) |  |
| Not available | 3 |  |  |  |  |  |  |  |  |  |  |  |  |  |  |  |

C: Cytoplasm

N: Nucleus

High: Immunostaining score > 3

^1^Linear-by-linear association

^2^Fisher exact test

^3^Pearson chi-square
