# Supplementary material for: Expression of CDK1Tyr15, pCDK1Thr161, Cyclin B1 (Total) and pCyclin B1Ser126 in Vulvar Squamous Cell Carcinoma and Their Relations with Clinicopatological Features and Prognosis
Source: PLoS One. 2015 Apr 7;10(4):e0121398. doi: 10.1371/journal.pone.0121398 (PMC4388712; doi:10.1371/journal.pone.0121398)
Supplement: S3 Table — (DOCX) [file pone.0121398.s003.docx]

**S3 Table.** Cyclin B1 (total) and pCyclin B1^Ser126^ expression in relation to clinicopathological variables

| **Variables** |  | **Cyclin B1 (total)** | | | | | | | |  | **pCyclin B1^Ser126^** | | | | | | |
| --- | --- | --- | --- | --- | --- | --- | --- | --- | --- | --- | --- | --- | --- | --- | --- | --- | --- |
|  |  | **(C)** | | |  | | **(N)** | | |  | **(C)** | | |  | **(N)** | | |
|  | **No.** | **High** | **(%)** | ***p*** |  | **High** | | **(%)** | ***p*** |  | **High** | **(%)** | ***p*** |  | **High** | **(%)** | ***p*** |
| Age |  |  |  | 0.980^1^ |  |  | |  | 0.315^1^ |  |  |  | 0.049^1^ |  |  |  | 0.041^1^ |
| 25-69 | 117 | 82 | (70) |  |  | 37 | | (32) |  |  | 36 | (31) |  |  | 37 | (32) |  |
| 70-84 | 146 | 102 | (70) |  |  | 36 | | (25) |  |  | 31 | (21) |  |  | 32 | (22) |  |
| 85+ | 34 | 24 | (71) |  |  | 9 | | (27) |  |  | 6 | (18) |  |  | 6 | (18) |  |
| FIGO |  |  |  | 0.286^2^ |  |  | |  | 0.099^2^ |  |  |  | 0.005^2^ |  |  |  | 0.007^2^ |
| Ia | 10 | 6 | (60) |  |  | 4 | | (40) |  |  | 4 | (40) |  |  | 4 | (40) |  |
| Ib | 137 | 88 | (64) |  |  | 33 | | (24) |  |  | 34 | (25) |  |  | 33 | (24) |  |
| II | 13 | 10 | (77) |  |  | 3 | | (23) |  |  | 2 | (15) |  |  | 2 | (15) |  |
| IIIa | 64 | 48 | (75) |  |  | 16 | | (25) |  |  | 16 | (25) |  |  | 17 | (27) |  |
| IIIb | 38 | 30 | (79) |  |  | 14 | | (37) |  |  | 5 | (13) |  |  | 6 | (16) |  |
| IIIc | 12 | 8 | (67) |  |  | 2 | | (17) |  |  | 2 | (17) |  |  | 2 | (17) |  |
| IVa | 5 | 3 | 60) |  |  | 1 | | (20) |  |  | 0 | (0) |  |  | 0 | (0) |  |
| IVb | 13 | 12 | (92) |  |  | 8 | | (62) |  |  | 9 | (69) |  |  | 9 | (69) |  |
| Not available | 5 |  |  |  |  |  | |  |  |  |  |  |  |  |  |  |  |
| Lymph node metastasis |  |  |  | 0.085^3^ |  |  | |  | 0.561^3^ |  |  |  | 0.437^3^ |  |  |  | 0.590^3^ |
| None | 164 | 107 | (65) |  |  | 42 | | (26) |  |  | 41 | (25) |  |  | 40 | (24) |  |
| Unilateral | 89 | 66 | (74) |  |  | 24 | | (27) |  |  | 23 | (26) |  |  | 24 | (27) |  |
| Bilateral | 38 | 31 | (82) |  |  | 13 | | (34) |  |  | 6 | (16) |  |  | 7 | (18) |  |
| Not available | 6 |  |  |  |  |  | |  |  |  |  |  |  |  |  |  |  |
| Tumor diameter (cm) |  |  |  | <0.001^1^ |  |  | |  | 0.003^1^ |  |  |  | 0.981^1^ |  |  |  | 0.993^1^ |
| 0.3-2.5 | 88 | 48 | (55) |  |  | 17 | | (19) |  |  | 22 | (25) |  |  | 23 | (26) |  |
| 2.6-4.0 | 93 | 68 | (73) |  |  | 19 | | (20) |  |  | 20 | (22) |  |  | 20 | (22) |  |
| 4.1-20.0 | 100 | 80 | (80) |  |  | 38 | | (38) |  |  | 25 | (25) |  |  | 26 | (26) |  |
| Not available | 16 |  |  |  |  |  | |  |  |  |  |  |  |  |  |  |  |
| Tumor differentiation |  |  |  | <0.001^3^ |  |  | |  | 0.003^3^ |  |  |  | 0.043^3^ |  |  |  | 0.032^3^ |
| Well | 73 | 35 | (48) |  |  | 11 | | (15) |  |  | 10 | (14) |  |  | 10 | (14) |  |
| Moderate | 153 | 111 | (73) |  |  | 42 | | (28) |  |  | 42 | (28) |  |  | 44 | (29) |  |
| Poor | 71 | 62 | (87) |  |  | 29 | | (41) |  |  | 21 | (30) |  |  | 21 | (30) |  |
| Depth of invasion (mm) |  |  |  | <0.001^1^ |  |  | |  | <0.001^1^ |  |  |  | 0.196^1^ |  |  |  | 0.249^1^ |
| 0.0-4.0 | 76 | 39 | (51) |  |  | 11 | | (15) |  |  | 21 | (28) |  |  | 21 | (28) |  |
| 4.1-8.0 | 98 | 67 | (86) |  |  | 23 | | (24) |  |  | 24 | (25) |  |  | 25 | (26) |  |
| 8.1-40.0 | 112 | 93 | (83) |  |  | 42 | | (38) |  |  | 22 | (20) |  |  | 23 | (21) |  |
| Not available | 11 |  |  |  |  |  | |  |  |  |  |  |  |  |  |  |  |
| Infiltration of vessel |  |  |  | 0.889^3^ |  |  | |  | 0.198^3^ |  |  |  | 0.964^3^ |  |  |  | 0.851^3^ |
| No | 229 | 160 | (70) |  |  | 59 | | (26) |  |  | 57 | (25) |  |  | 59 | (26) |  |
| Yes | 65 | 46 | (71) |  |  | 22 | | (34) |  |  | 16 | (25) |  |  | 16 | (25) |  |
| Not available | 3 |  |  |  |  |  | |  |  |  |  |  |  |  |  |  |  |

C: Cytoplasm

N: Nucleus

High: Immunostaining score > 3

^1^Linear-by-linear association

^2^Fisher exact test

^3^Pearson chi-square
