# Supplementary material for: Expression of CDK1Tyr15, pCDK1Thr161, Cyclin B1 (Total) and pCyclin B1Ser126 in Vulvar Squamous Cell Carcinoma and Their Relations with Clinicopatological Features and Prognosis
Source: PLoS One. 2015 Apr 7;10(4):e0121398. doi: 10.1371/journal.pone.0121398 (PMC4388712; doi:10.1371/journal.pone.0121398)
Supplement: S4 Table — (DOCX) [file pone.0121398.s004.docx]

**S4 Table.** CDK1^Tyr15^ and pCDK1^Thr161^ expression in relation to cell cycle proteins and HPV

| **Variables** |  | **CDK1^Tyr15^** | | | | | | |  | **pCDK1^Thr161^** | | | | | | |
| --- | --- | --- | --- | --- | --- | --- | --- | --- | --- | --- | --- | --- | --- | --- | --- | --- |
|  |  | **(C)** | | |  | **(N)** | | |  | **(C)** | | |  | **(N)** | | |
|  | **No.** | **High** | **(%)** | ***p^1^*** |  | **High** | **(%)** | ***p^1^*** |  | **High** | **(%)** | ***p^1^*** |  | **High** | **(%)** | ***p^1^*** |
| CDK1^Tyr15^ (C) |  |  |  | - |  |  |  | - |  |  |  | <0.001 |  |  |  | <0.001 |
| Low (< 3) | 102 | - | - |  |  | - | - |  |  | 13 | (13) |  |  | 64 | (63) |  |
| High (≥ 3) | 195 | - | - |  |  | - | - |  |  | 84 | (43) |  |  | 167 | (86) |  |
| CDK1^Tyr15^ (N) |  |  |  | - |  |  |  | - |  |  |  | 0.036 |  |  |  | 0.004 |
| Low (< 3) | 219 | - | - |  |  | - | - |  |  | 64 | (29) |  |  | 161 | (74) |  |
| High (≥ 3) | 78 | - | - |  |  | - | - |  |  | 33 | (43) |  |  | 70 | (90) |  |
| pCDK1^Thr161^ (C) |  |  |  | <0.001 |  |  |  | 0.036 |  |  |  | - |  |  |  | - |
| Low (< 3) | 200 | 111 | (56) |  |  | 45 | (23) |  |  | - | - |  |  | - | - |  |
| High (≥ 3) | 97 | 84 | (87) |  |  | 33 | (34) |  |  | - | - |  |  | - | - |  |
| pCDK1^Thr161^ (N) |  |  |  | <0.001 |  |  |  | 0.004 |  |  |  | - |  |  |  | - |
| Low (< 3) | 66 | 28 | (42) |  |  | 8 | (12) |  |  | - | - |  |  | - | - |  |
| High (≥ 3) | 231 | 167 | (72) |  |  | 70 | (30) |  |  | - | - |  |  | - | - |  |
| Cyclin B1(total) (C) |  |  |  | <0.001 |  |  |  | 0.021 |  |  |  | <0.001 |  |  |  | <0.001 |
| Low (< 3) | 89 | 25 | (28) |  |  | 15 | (17) |  |  | 7 | (8) |  |  | 56 | (63) |  |
| High (≥ 3) | 208 | 170 | (82) |  |  | 63 | (30) |  |  | 90 | (43) |  |  | 175 | (84) |  |
| Cyclin B1(total) (N) |  |  |  | <0.001 |  |  |  | <0.001 |  |  |  | <0.001 |  |  |  | 0.012 |
| Low (< 3) | 215 | 124 | (58) |  |  | 43 | (20) |  |  | 57 | (27) |  |  | 159 | (74) |  |
| High (≥ 3) | 82 | 71 | (87) |  |  | 35 | (43) |  |  | 40 | (49) |  |  | 72 | (88) |  |
| pCyclin B1^Ser126^ (C) |  |  |  | <0.001 |  |  |  | <0.001 |  |  |  | <0.001 |  |  |  | 0.001 |
| Low (< 3) | 224 | 129 | (58) |  |  | 44 | (20) |  |  | 60 | (27) |  |  | 164 | (73) |  |
| High (≥ 3) | 73 | 66 | (90) |  |  | 34 | (47) |  |  | 37 | (51) |  |  | 67 | (92) |  |
| pCyclin B1^Ser126^ (N) |  |  |  | <0.001 |  |  |  | <0.001 |  |  |  | <0.001 |  |  |  | 0.001 |
| Low (< 3) | 222 | 127 | (57) |  |  | 44 | (20) |  |  | 59 | (27) |  |  | 162 | (73) |  |
| High (≥ 3) | 75 | 68 | (91) |  |  | 34 | (45) |  |  | 38 | (51) |  |  | 69 | (92) |  |
| 14-3-3σ (N) |  |  |  | 0.699 |  |  |  | 0.013 |  |  |  | 0.896 |  |  |  | 0.025 |
| Low (= 0) | 121 | 81 | (67) |  |  | 41 | (34) |  |  | 39 | (32) |  |  | 102 | (84) |  |
| High (> 0) | 176 | 114 | (65) |  |  | 37 | (21) |  |  | 58 | (33) |  |  | 129 | (73) |  |
| 14-3-3β (C) |  |  |  | 0.002 |  |  |  | 0.995 |  |  |  | 0.556 |  |  |  | 0.618 |
| Low (≤ 1) | 61 | 30 | (49) |  |  | 16 | (26) |  |  | 18 | (30) |  |  | 46 | (75) |  |
| High (> 1) | 236 | 165 | (70) |  |  | 62 | (26) |  |  | 79 | (31) |  |  | 185 | (78) |  |
| 14-3-3γ (C) |  |  |  | 0.006 |  |  |  | 0.825 |  |  |  | 0.479 |  |  |  | 0.530 |
| Low (≤ 3) | 125 | 71 | (57) |  |  | 32 | (26) |  |  | 38 | (30) |  |  | 95 | (76) |  |
| High (> 3) | 172 | 124 | (72) |  |  | 46 | (27) |  |  | 59 | (34) |  |  | 136 | (79) |  |
| 14-3-3η (C) |  |  |  | <0.001 |  |  |  | 0.166 |  |  |  | 0.132 |  |  |  | 0.004 |
| Low (≤ 3) | 138 | 72 | (52) |  |  | 31 | (23) |  |  | 39 | (28) |  |  | 97 | (70) |  |
| High (> 3) | 159 | 123 | (77) |  |  | 47 | (30) |  |  | 58 | (37) |  |  | 134 | (84) |  |
| 14-3-3ε (N) |  |  |  | 0.194 |  |  |  | 0.006 |  |  |  | 0.219 |  |  |  | 0.029 |
| Low (= 0) | 201 | 127 | (63) |  |  | 43 | (21) |  |  | 61 | (30) |  |  | 149 | (74) |  |
| High (> 0) | 96 | 68 | (71) |  |  | 35 | (37) |  |  | 36 | (38) |  |  | 82 | (85) |  |
| CDC25A (N) |  |  |  | 0.346 |  |  |  | 0.001 |  |  |  | 0.032 |  |  |  | 0.121 |
| Low (≤ 6) | 146 | 92 | (63) |  |  | 26 | (18) |  |  | 39 | (27) |  |  | 108 | (74) |  |
| High (> 6) | 151 | 103 | (68) |  |  | 52 | (34) |  |  | 58 | (38) |  |  | 123 | (82) |  |
| CDC25B (N) |  |  |  | <0.001 |  |  |  | 0.002 |  |  |  | 0.055 |  |  |  | 0.089 |
| Low (≤ 6) | 250 | 152 | (61) |  |  | 57 | (23) |  |  | 76 | (30) |  |  | 190 | (76) |  |
| High (> 6) | 47 | 43 | (92) |  |  | 21 | (45) |  |  | 21 | (45) |  |  | 41 | (87) |  |
| CDC25C (C) |  |  |  | 0.068 |  |  |  | 0.006 |  |  |  | 0.005 |  |  |  | 0.002 |
| Low (≤ 3) | 110 | 65 | (59) |  |  | 39 | (36) |  |  | 25 | (23) |  |  | 75 | (68) |  |
| High (> 3) | 187 | 130 | (70) |  |  | 39 | (21) |  |  | 72 | (39) |  |  | 156 | (83) |  |
| pCDC25C^Ser216^ (C) |  |  |  | <0.001 |  |  |  | 0.081 |  |  |  | <0.001 |  |  |  | 0.004 |
| Low (≤ 3) | 147 | 76 | (52) |  |  | 32 | (22) |  |  | 24 | (16) |  |  | 104 | (71) |  |
| High (> 3) | 150 | 119 | (79) |  |  | 46 | (31) |  |  | 73 | (49) |  |  | 127 | (85) |  |
| Wee1 (C) |  |  |  | 0.053 |  |  |  | 0.208 |  |  |  | 0.016 |  |  |  | <0.001 |
| Low (≤ 0) | 140 | 84 | (60) |  |  | 32 | (23) |  |  | 36 | (26) |  |  | 96 | (69) |  |
| High (> 0) | 157 | 111 | (71) |  |  | 46 | (29) |  |  | 61 | (39) |  |  | 135 | (86) |  |
| Wee1 (N) |  |  |  | 0.004 |  |  |  | <0.001 |  |  |  | 0.012 |  |  |  | <0.001 |
| Low (≤ 6) | 220 | 134 | (61) |  |  | 42 | (19) |  |  | 63 | (29) |  |  | 159 | (72) |  |
| High (> 6) | 77 | 61 | (79) |  |  | 36 | (47) |  |  | 34 | (44) |  |  | 72 | (94) |  |
| HPV^2^ |  |  |  | 0.017 |  |  |  | <0.001 |  |  |  | 0.015 |  |  |  | 0.097 |
| Low (-) | 166 | 96 | (58) |  |  | 23 | (14) |  |  | 41 | (25) |  |  | 121 | (73) |  |
| High (+) | 41 | 32 | (78) |  |  | 18 | (44) |  |  | 18 | (44) |  |  | 35 | (85) |  |
| Not available | 90 |  |  |  |  |  |  |  |  |  |  |  |  |  |  |  |

C: Cytoplasm

N: Nucleus

High: Immunostaining score > 3

^1^Pearson chi-square

^2^In previous report [36]
