# Supplementary material for: Expression of CDK1Tyr15, pCDK1Thr161, Cyclin B1 (Total) and pCyclin B1Ser126 in Vulvar Squamous Cell Carcinoma and Their Relations with Clinicopatological Features and Prognosis
Source: PLoS One. 2015 Apr 7;10(4):e0121398. doi: 10.1371/journal.pone.0121398 (PMC4388712; doi:10.1371/journal.pone.0121398)
Supplement: S5 Table — (DOCX) [file pone.0121398.s005.docx]

**S5 Table.** Cyclin B1 (total) and pCyclin B1^Ser126^ expression in relation to cell cycle proteins and HPV

| **Variables** |  | **Cyclin B1(total)** | | | | | | |  | **pCyclin B1^Ser126^** | | | | | | |
| --- | --- | --- | --- | --- | --- | --- | --- | --- | --- | --- | --- | --- | --- | --- | --- | --- |
|  |  | **(C)** | | |  | **(N)** | | |  | **(C)** | | |  | **(N)** | | |
|  | **No.** | **High** | **(%)** | ***p^1^*** |  | **High** | **(%)** | ***p^1^*** |  | **High** | **(%)** | ***p^1^*** |  | **High** | **(%)** | ***p^1^*** |
| Cyclin B1(total) (C) |  |  |  | - |  |  |  | - |  |  |  | <0.001 |  |  |  | <0.001 |
| Low (< 3) | 89 | - | - |  |  | - | - |  |  | 5 | (6) |  |  | 5 | (6) |  |
| High (≥ 3) | 208 | - | - |  |  | - | - |  |  | 68 | (33) |  |  | 71 | (34) |  |
| CyclinB1(total) (N) |  |  |  | - |  |  |  | - |  |  |  | <0.001 |  |  |  | <0.001 |
| Low (< 3) | 215 | - | - |  |  | - | - |  |  | 31 | (14) |  |  | 32 | (15) |  |
| High (≥ 3) | 82 | - | - |  |  | - | - |  |  | 42 | (51) |  |  | 44 | (54) |  |
| pCyclin B1^Ser126^ (C) |  |  |  | <0.001 |  |  |  | <0.001 |  |  |  | - |  |  |  | - |
| Low (< 3) | 224 | 140 | (63) |  |  | 40 | (18) |  |  | - | - |  |  | - | - |  |
| High (≥ 3) | 73 | 68 | (93) |  |  | 42 | (58) |  |  | - | - |  |  | - | - |  |
| pCyclin B1^Ser126^ (N) |  |  |  | <0.001 |  |  |  | <0.001 |  |  |  | - |  |  |  | - |
| Low (< 3) | 222 | 138 | (62) |  |  | 38 | (17) |  |  | - | - |  |  | - | - |  |
| High (≥ 3) | 75 | 70 | (93) |  |  | 44 | (59) |  |  | - | - |  |  | - | - |  |
| 14-3-3σ (N) |  |  |  | <0.001 |  |  |  | 0.091 |  |  |  | 0.730 |  |  |  | 0.695 |
| Low (= 0) | 121 | 70 | (58) |  |  | 27 | (22) |  |  | 31 | (26) |  |  | 32 | (26) |  |
| High (> 0) | 176 | 138 | (78) |  |  | 55 | (31) |  |  | 42 | (24) |  |  | 43 | (24) |  |
| 14-3-3γ (C) |  |  |  | <0.001 |  |  |  | 0.025 |  |  |  | 0.940 |  |  |  | 0.906 |
| Low (≤ 3) | 125 | 72 | (58) |  |  | 26 | (21) |  |  | 31 | (25) |  |  | 32 | (26) |  |
| High (> 3) | 172 | 136 | (79) |  |  | 56 | (33) |  |  | 42 | (24) |  |  | 43 | (25) |  |
| 14-3-3ζ (C) |  |  |  | <0.001 |  |  |  | 0.003 |  |  |  | 0.760 |  |  |  | 0.571 |
| Low (≤ 3) | 147 | 87 | (59) |  |  | 29 | (20) |  |  | 35 | (24) |  |  | 35 | (24) |  |
| High (> 3) | 150 | 121 | (81) |  |  | 53 | (35) |  |  | 38 | (25) |  |  | 40 | (27) |  |
| 14-3-3η (C) |  |  |  | <0.001 |  |  |  | 0.002 |  |  |  | 0.007 |  |  |  | 0.018 |
| Low (≤ 3) | 138 | 74 | (54) |  |  | 26 | (19) |  |  | 24 | (17) |  |  | 26 | (19) |  |
| High (> 3) | 159 | 134 | (84) |  |  | 56 | (35) |  |  | 49 | (31) |  |  | 49 | (31) |  |
| 14-3-3ε (N) |  |  |  | 0.118 |  |  |  | 0.071 |  |  |  | 0.003 |  |  |  | 0.005 |
| Low (= 0) | 201 | 135 | (67) |  |  | 49 | (24) |  |  | 39 | (19) |  |  | 41 | (20) |  |
| High (> 0) | 96 | 73 | (76) |  |  | 33 | (34) |  |  | 34 | (35) |  |  | 34 | (35) |  |
| CDC25B (N) |  |  |  | 0.005 |  |  |  | 0.001 |  |  |  | 0.017 |  |  |  | 0.025 |
| Low (≤ 6) | 250 | 167 | (67) |  |  | 60 | (24) |  |  | 55 | (22) |  |  | 57 | (23) |  |
| High (> 6) | 47 | 41 | (87) |  |  | 22 | (47) |  |  | 18 | (38) |  |  | 18 | (38) |  |
| pCDC25C^Ser216^ (C) |  |  |  | <0.001 |  |  |  | <0.001 |  |  |  | 0.001 |  |  |  | 0.001 |
| Low (≤ 3) | 147 | 83 | (57) |  |  | 22 | (15) |  |  | 24 | (16) |  |  | 25 | (17) |  |
| High (> 3) | 150 | 125 | (83) |  |  | 60 | (40) |  |  | 49 | (33) |  |  | 50 | (33) |  |
| Wee1 (C) |  |  |  | 0.022 |  |  |  | 0.084 |  |  |  | 0.001 |  |  |  | <0.001 |
| Low (≤ 0) | 140 | 89 | (64) |  |  | 32 | (23) |  |  | 22 | (16) |  |  | 21 | (15) |  |
| High (> 0) | 157 | 119 | (76) |  |  | 50 | (32) |  |  | 51 | (33) |  |  | 54 | (34) |  |
| Wee1 (N) |  |  |  | 0.009 |  |  |  | 0.010 |  |  |  | <0.001 |  |  |  | <0.001 |
| Low (≤ 6) | 220 | 145 | (66) |  |  | 52 | (24) |  |  | 42 | (19) |  |  | 44 | (20) |  |
| High (> 6) | 77 | 63 | (82) |  |  | 30 | (39) |  |  | 31 | (40) |  |  | 31 | (40) |  |
| HPV^2^ |  |  |  | 0.001 |  |  |  | 0.001 |  |  |  | 0.003 |  |  |  | 0.001 |
| Low (-) | 166 | 106 | (64) |  |  | 32 | (19) |  |  | 29 | (18) |  |  | 30 | (18) |  |
| High (+) | 41 | 37 | (90) |  |  | 18 | (44) |  |  | 16 | (39) |  |  | 17 | (42) |  |
| Not available | 90 |  |  |  |  |  |  |  |  |  |  |  |  |  |  |  |

C: Cytoplasm

N: Nucleus

High: Immunostaining score > 3

^1^Pearson chi-square

^2^In previous report [36]
